# Potential‐Controlled Ammonium‐Assisted Reversible Electrodeposition of Polyoxometalates for Hybrid Redox Flow Batteries

**DOI:** 10.1002/advs.77673

**Published:** 2026-09-16

**Authors:** Ke Wang, Nikhil Arya, Sadia Shahid, Montaha Anjass

**Affiliations:** ^1^ Institute of Inorganic Chemistry I Ulm University Ulm Germany; ^2^ Department of Chemistry University of Sharjah Sharjah UAE; ^3^ CELEST Green Energy Lab Ulm Ulm University Ulm Germany

**Keywords:** ammonium‐assisted electrodeposition, hybrid redox flow battery, molecular energy storage, polyoxometalates

## Abstract

Counter‐cations play a key role in modulating the redox chemistry and phase behavior of polyoxometalates (POMs). In this study, we report a potential‐controlled, ammonium‐assisted electrodeposition of POMs from nonaqueous electrolytes. Owing to its tetrahedral geometry and hydrogen‐bonding capability, NH_4_^+^ uniquely stabilizes reduced POM clusters at the electrode interface, enabling reversible solid‐to‐solution transitions. Using the mixed‐valence vanadium cluster [V_18_O_46_(NO_3_)]^5−^ and Keggin‐type POM cluster, [PMo_12_O_40_]^3−^ as model systems, we demonstrate controlled reversible charge‐transfer dynamics, including effective mass deposition under reductive potentials and complete dissolution upon oxidation, as confirmed by cyclic voltammetry and electrochemical quartz crystal microbalance (EQCM) measurements. Microscopic and spectroscopic investigations reveal that electrodeposition at low potentials involves partial reduction of redox‐active metal centers within the POM framework, accompanied by NH_4_^+^ incorporation via hydrogen‐bonded networks, forming NH_4_^+^‐coupled POM‐derived deposits, which exhibit a significantly different pathway from the classic metal electrodeposition (metal plating). Leveraging this reversible behavior, we propose the design of POM electrodeposition‐based hybrid redox flow battery (RFB), where NH_4_^+^‐driven POM electrodeposition/dissolution in the negative half‐cell enables electron storage/release during charge/discharge processes, and validate the design through proof‐of‐concept battery demonstrations. These findings provide important initial insights into NH_4_^+^‐driven reversible POM electrodeposition and highlight its potential in advancing electrochemical energy storage technologies.

## Graphical abstract

Potential‐controlled NH_4_^+^‐assisted reversible electrodeposition of molecular metal oxide clusters enables dynamic solution‐to‐solid electron storage. Leveraging this deposition/dissolution reaction chemistry, polyoxometalate‐based hybrid flow batteries are demonstrated as a new platform for deposition‐based electrochemical energy storage.

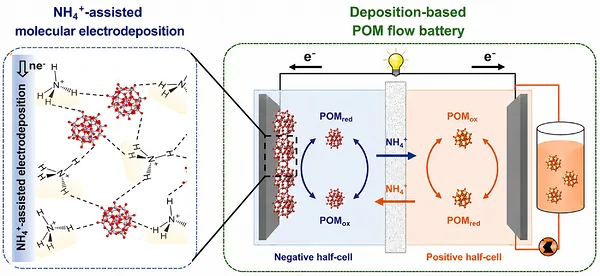

## 1 Introduction

Polyoxometalates (POMs) are a class of anionic molecular metal oxides that contain multiple high‐valent metal centers (such as Mo, W and V) and feature rich electrochemistry [1, 2]. Counter‐cations are crucial in POM (electro‐)chemistry, beyond the simple role of charge balance [3]. During the reduction process of a POM cluster in an electrolyte solution, in addition to pristine counter‐cations, the cations from the supporting electrolyte also interact with the reduced POM cluster to maintain electroneutrality, which can influence the redox behavior [4, 5, 6, 7]. Matson and co‐workers investigated how different cations affect the redox behavior of the polyoxovanadate (POV)‐alkoxide cluster ([V_6_O_7_(OCH)_3_]). They found that in metal trifluoromethanesulfonate (MOTf)/MeCN solutions (M = Li^+^, Na^+^, K^+^), the most reducing wave of the cluster shifted anodically compared to (nBu_4_N)OTf/MeCN. This shift followed the order K^+^ < Na^+^ < Li^+^, indicating a stronger anodic shift with smaller cations [8]. This trend was attributed to the nature of the cation‐POM interaction modes: Li^+^ binds selectively to the cluster, while the K^+^ exhibits weaker, non‐selective interactions. Recently, our group reported a 4.9‐electron storage capability in a POV cluster, K_5_(CH_3_CN)_3_[V_12_O_32_Cl], via the electrodeposition from an (*n*Bu_4_N)PF_6_/MeCN solution under a reduction potential of −500 mV (*vs*. Ag^+^/Ag) [9]. In addition, the reduction of an analogous cluster, (*n*Bu_4_N)_3_(H_2_NMe_2_)_2_MeCN[V_12_O_32_Cl], in a KPF_6_/MeCN solution also led to electrodeposition behavior; however, this phenomenon was not observed in an (*n*Bu_4_N)PF_6_/MeCN solution, indicating the essential role of K^+^ in the POM electrodeposition. In addition to potassium–ion electrolyte solutions, POM‐based electrodeposition from lithium–ion and sodium–ion electrolyte solutions has also been investigated [10, 11]. Notably, the potential‐driven electrodeposition of POMs from POM‐containing electrolyte solutions is fundamentally distinct from conventional metal electrodeposition (also called metal plating). Rather than forming zero‐valent metallic deposits, the deposition process is driven by cation‐POM interactions under electrochemical reduction, resulting in reversible cation‐coupled metal oxide deposits on the electrode surface.

Unlike conventional metallic cations, NH_4_^+^ is a non‐metallic, polyatomic ion with directional hydrogen‐bonding capability [12], and is considered a promising charge carrier due to its abundant resources, relatively low molecular mass (18 g mol^−1^), and fast diffusion kinetics [13]. Up to now, little work on electrodeposition from ammonium electrolyte solutions has been reported. Quaternary alkyl ammonium cations were reported to impede metal electrodeposition and the long‐chain cations tend to inhibit the salts from crystallizing [3, 14]. Since NH_4_^+^ and K^+^ exhibit similar ionic radii, NH_4_^+^‐assisted POM electrodeposition under reduction potentials can be anticipated [9, 13]. Importantly, beyond electrostatic charge compensation, NH_4_^+^ can engage in directional hydrogen‐bonding interactions with oxygen‐rich frameworks, which have been revealed between NH_4_^+^ and transition metal oxides [15], suggesting that analogous interactions may also form between NH_4_^+^ and molecular metal oxides such as POMs [16]. Since hydrogen bonding interactions can promote the solubility in solutions [17] and stabilize solid‐state structures with improved electrochemical performance [18], NH_4_^+^ may uniquely stabilize reduced POM clusters at electrode/electrolyte interface, thereby promoting reversible POM‐derived electrodeposition from solution rather than conventional zero‐valent metal plating. Consequently, the charge‐transfer reactions of POM clusters in an ammonium‐based electrolyte are particularly promising for reversible electrochemical energy‐storage applications.

Inspired by the above hypothesis, herein, we exploit non‐metallic NH_4_^+^ to direct the solution‐based redox behaviors of POM clusters. A mixed‐valence POM cluster, [V_18_O_46_(NO_3_)]^5−^ (= **{V_18_}**), and a fully‐oxidized Keggin‐type POM cluster, [PMo_12_O_40_]^3−^ (= **{PMo_12_}**), were investigated in a nonaqueous ammonium electrolyte solution. Electrochemical studies showed reversible mass deposition/dissolution within selected potential ranges. Based on the NH_4_^+^‐assisted POM electrodeposition, proof‐of‐concept POM hybrid RFBs were demonstrated.

## 2 Results and Discussion

### 2.1 Cyclic Voltammetry

**FIGURE 1:**
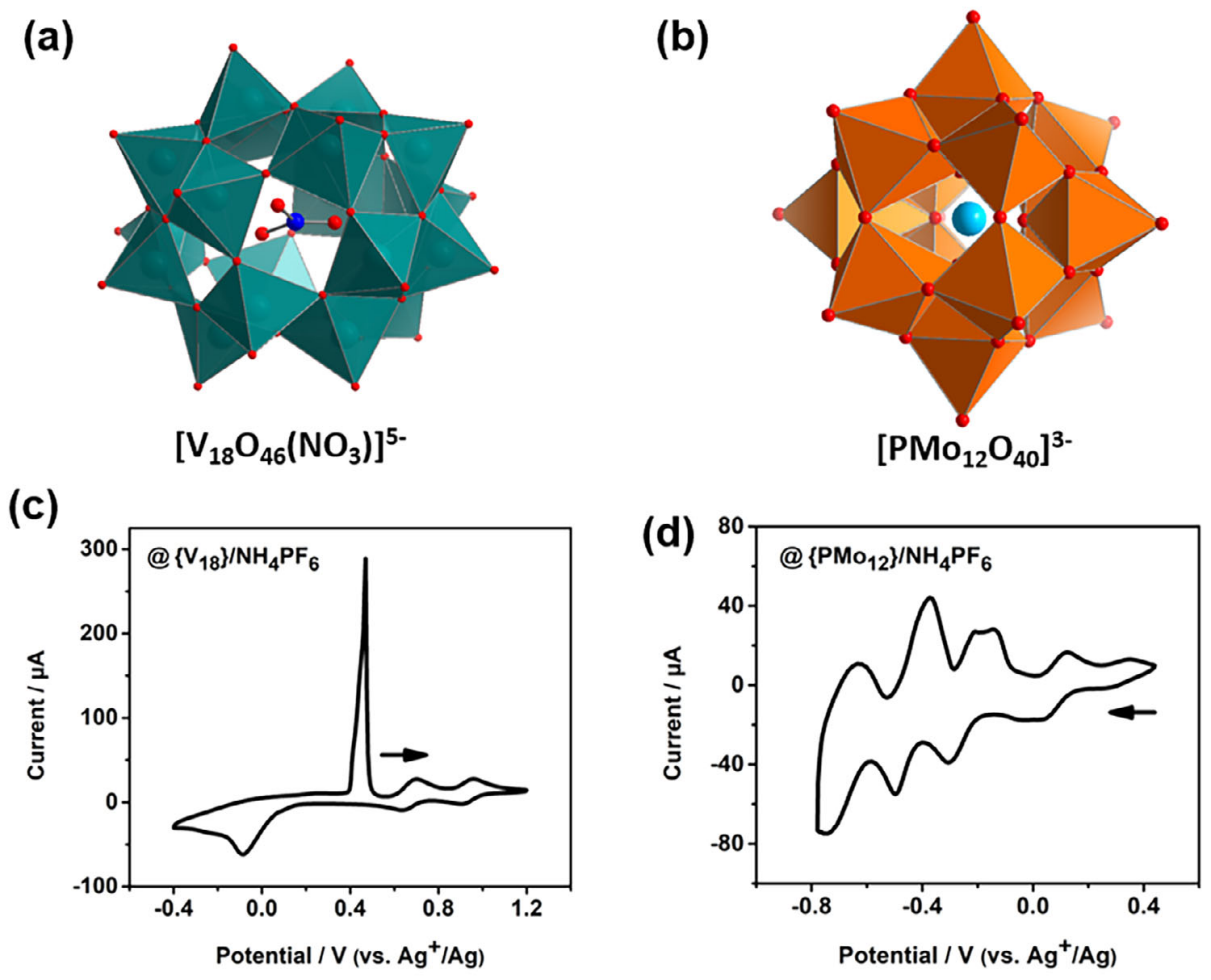
Molecular structure of (a) [V_18_O_46_(NO_3_)]^5−^ and (b) [PMo_12_O_40_]^3−^, color scheme: oxygen (red), nitrogen (blue), vanadium atoms and [VO_5_] polyhedral (teal), phosphorus (cyan), molybdenum atoms and [MoO_6_] polyhedral (orange). CV response of (c) [V_18_O_46_(NO_3_)]^5−^ and (d) [PMo_12_O_40_]^3−^ on glassy carbon (Ø = 3 mm) in NH_4_PF_6_/MeCN (experimental conditions: anhydrous, de‐aerated acetonitrile containing concentrated NH_4_PF_6_ as supporting salt, [V_18_O_46_(NO_3_)]^5−^ or [PMo_12_O_40_]^3−^ = 1 mm, scan rate: 50 mV s^−1^).

As demonstrated in our previous study [9], controlled electrochemical reduction of the POV cluster induces reversible K^+^‐assisted electrodeposition, leading to the formation of a thin film on the electrode surface. Here, we extend this approach to non‐metallic tetrahedral NH_4_^+^ and other polyoxometalate clusters. The redox behaviors of two representative POM clusters, [V_18_O_46_(NO_3_)]^5−^ and [PMo_12_O_40_]^3−^, were initially investigated by cyclic voltammetry (CV) on a glassy carbon working electrode (Ø = 3 mm). The nitrate‐templated [V_18_O_46_(NO_3_)]^5−^ cluster (Figure 1a, synthesized according to reported literature [19]) exhibited five pairs of redox waves in an (*n*Bu_4_N)PF_6_/MeCN solution within the potential range from −1300 to +1300 mV (vs. Ag^+^/Ag), corresponding to −1669 to +931 mV (vs. Fc^+^/Fc) after potential calibration, including three waves in reduction direction and two waves in oxidation direction (Figure S1a). By comparison, in an NH_4_PF_6_/MeCN solution, the CV response in the reduction direction exhibited a single broad reduction peak as the potential was lowered to −400 mV (vs. Ag^+^/Ag), corresponding to −690 mV (vs. Fc^+^/Fc) (Figure 1c).

No additional reduction waves were observed when the potential was further lowered. During the subsequent oxidative sweep, a sharp oxidation peak with a high peak current appeared at +470 mV (vs. Ag^+^/Ag, corresponding to +180 mV (vs. Fc^+^/Fc)), indicating efficient reoxidation of the reduced species and facilitated electron release. The distinct redox behavior of [V_18_O_46_(NO_3_)]^5−^ in NH_4_PF_6_/MeCN solution in the low‐potential region reflects fundamentally different cation‐POV interactions compared with (*n*Bu_4_N)PF_6_ electrolyte. In addition, the well‐defined reduction/oxidation peaks can appear under repeated CV scans, suggesting that the **{V_18_}‐**POV cluster reversibly interconverts between the initial [V_18_O_46_(NO_3_)]^5−^ nanocluster and a stable reduced form in the presence of NH_4_^+^. In addition, at higher anodic potentials, two well‐separated (quasi‐)reversible redox waves were retained in the NH_4_PF_6_/MeCN solution at approximately +700 and +960 mV (*vs*. Ag^+^/Ag), corresponding to +410 and +670 mV (*vs*. Fc^+^/Fc), respectively, identical to those observed for the **{V_18_}** cluster in (*n*Bu_4_N)PF_6_‐based electrolyte. These characteristic anodic processes are in line with the mixed‐valence **{V_18_}** and indicate that, at higher potentials than the “open‐circuit potential”, these two solution‐based charge‐transfer processes were well preserved in the presence of NH_4_^+^, and remain essentially unaffected by electrolyte cation [19, 20]. Although these anodic waves may also include contributions from dissolved **{V_18_}** clusters in the bulk electrolyte, the absence of additional oxidation features in subsequent cycles is consistent with the preservation of the characteristic electrochemical response of **{V_18_}‐**derived species within the investigated potential window.

For the fully‐oxidized POM cluster, the Keggin‐type [PMo_12_O_40_]^3−^ (Figure 1b) comprises twelve octahedral MoO_6_ units surrounding the central phosphate template and is capable of multi‐electron storage by successive reduction of the Mo^VI^ centers [21]. The cation effect on the voltammetric behavior of the [PMo_12_O_40_]^3−^ cluster in nonaqueous media has been previously investigated [22, 23]. In MeCN, three one‐electron redox waves appear in the presence of (*n*Bu_4_N)^+^ within the potential range from −1300 to 300 mV (vs. Ag^+^/Ag, corresponding to −1669 to −69 mV (vs. Fc^+^/Fc)), whereas replacing the supporting cation with Li^+^ results in merging of two one‐electron waves into a single two‐electron wave [22, 23]. In this work, a comparable set of three redox waves was also observed for [PMo_12_O_40_]^3−^ in (*n*Bu_4_N)PF_6_/MeCN within a similar potential range (Figure S1b). Notably, upon extending the cathodic potential window beyond the range reported in previous studies, two additional redox waves were observed, revealing previously unreported low‐potential redox activity of [PMo_12_O_40_]^3−^ cluster in an (*n*Bu_4_N)^+^‐based electrolyte. To investigate the redox behavior of [PMo_12_O_40_]^3−^ cluster in the presence of NH_4_^+^, the supporting electrolyte salt was replaced with NH_4_PF_6_. Under these conditions, the [PMo_12_O_40_]^3−^ cluster exhibited several stepwise redox waves as the potential was lowered to −780 mV (vs. Ag^+^/Ag, corresponding to −1070 mV (vs. Fc^+^/Fc)) (Figure 1d). Upon extending the potential window to more negative values, additional cathodic peaks with higher current values appeared (Figure S2); however, the redox behavior became irreversible. Compared with (*n*Bu_4_N)^+^ and alkali‐metal cations, the significantly different redox behavior of [PMo_12_O_40_]^3−^ cluster in the presence of NH_4_^+^ indicates different NH_4_^+^‐POM interactions, suggesting a stronger coupling between ammonium cations and the reduced molecular oxide framework.

The results discussed above demonstrate that the non‐metallic NH_4_^+^ cation enables distinct interactions with the anionic [V_18_O_46_(NO_3_)]^5−^ and [PMo_12_O_40_]^3−^ species. While both POM/NH_4_^+^ electrolyte systems exhibit pronounced cation‐dependent redox responses under lower potentials than the “open‐circuit potential”, the [PMo_12_O_40_]^3−^ cluster exhibited more complex redox behavior in the ammonium‐based electrolyte than the [V_18_O_46_(NO_3_)]^5−^ cluster. Notably, compared with the bare and unchanged surface of the working electrode during the CV tests in the (*n*Bu_4_N)PF_6_/MeCN solution, a patchy and rough surface was observed on the electrode surface when the electrolyte was changed to NH_4_PF_6_/MeCN, suggesting possible electrodeposition behavior [9, 24]. This was further investigated in detail via the highly sensitive electrochemical quartz crystal microbalance (EQCM) technique and is discussed in the following section.

### 2.2 Electrochemical Quartz Microbalance

**FIGURE 2:**
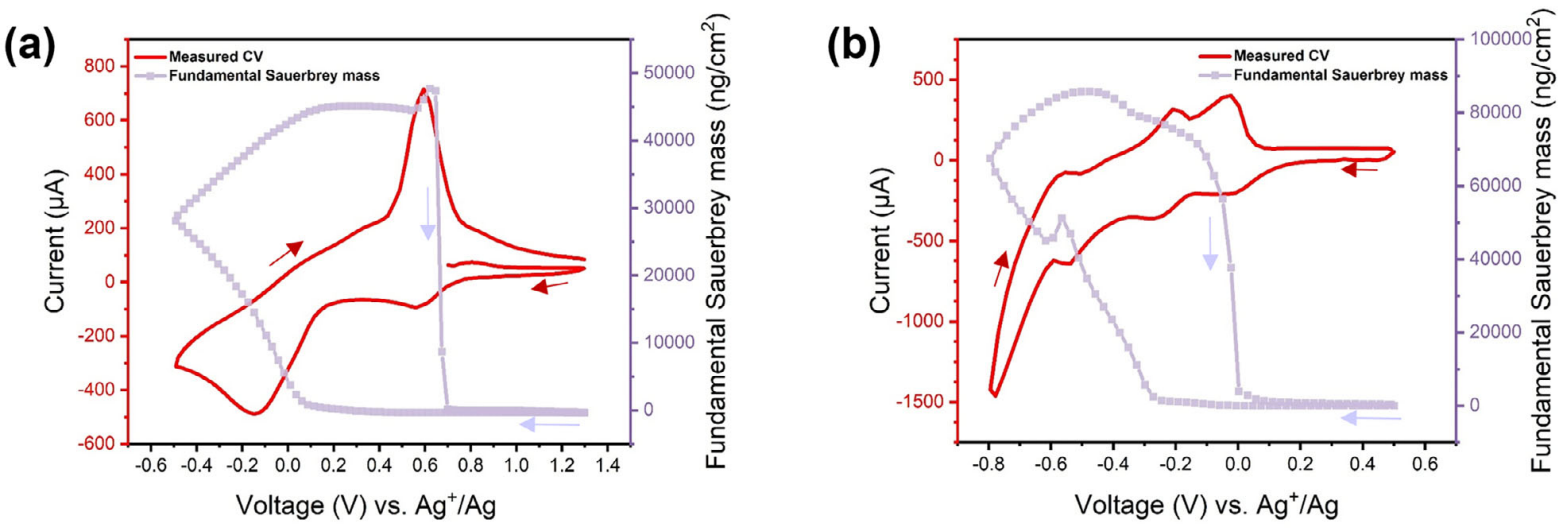
EQCM measurements and CV responses of (a) [V_18_O_46_(NO_3_)]^5−^ cluster in NH_4_PF_6_/MeCN in the potential range from −500 to +1300 mV (vs. Ag^+^/Ag), and (b) [PMo_12_O_40_]^3−^ cluster in NH_4_PF_6_/MeCN in the potential range from −800 to +500 mV (vs. Ag^+^/Ag), respectively, (experimental conditions: anhydrous, de‐aerated acetonitrile containing concentrated NH_4_PF_6_ as supporting salt, [V_18_O_46_(NO_3_)]^5−^ or [PMo_12_O_40_]^3−^ = 0.5 mm, scan rate: 30 mV s^−1^).

To obtain mechanistic insight into the NH_4_^+^‐assisted deposition/dissolution process, the electrochemical responses were investigated using electrochemical quartz crystal microbalance (EQCM), which simultaneously monitors charge transfer and mass variations at the electrode/electrolyte interface. In these experiments, the mass variation was monitored on a 12 mm gold‐coated quartz crystal (see ESI Section 4 for experimental details). The EQCM frequency response was correlated with CV responses to quantify mass variations and fluctuations at the surface of the working electrode according to the Sauerbrey relation (Figure 2). The [V_18_O_46_(NO_3_)]^5−^ cluster was examined within a potential window from −500 to +1300 mV (vs. Ag^+^/Ag), as illustrated in Figure 2a. During the initial anodic scan, negligible mass change on the electrode surface was detected, indicating that the POM species during the charge‐transfer reactions in this potential range remained dissolved in the solution phase. Significant mass variation began at approximately 0 mV (vs. Ag^+^/Ag) when the potential was scanned in the reduction direction, confirming the substantial mass deposition on the electrode surface, which aligned with the findings from the previous potassium‐mediated electrodeposition studies [9]. The mass deposition further increased during the following oxidative sweep and reached the maximum value when the highest oxidation current was reached, followed by a rapid mass decrease returning to the initial mass value [9, 21]. The complete mass extraction from the electrode surface suggested a rapid dissolution event [21]. The EQCM measurements directly visualize the dynamic solution‐to‐solid transition. Initially, the POM clusters remain fully dissolved in the NH_4_PF_6_/MeCN electrolyte. Upon electrochemical reduction, insoluble NH_4_^+^‐coupled POM‐derived species deposited onto the conductive substrates, resulting in a progressive increase in deposited mass. Under sufficient oxidation potentials, the reversed process occurred, leading to dissolution of the deposited POM‐derived phase and the return of the redox active species into the solution phase.

To assess the electrochemical stability of the [V_18_O_46_(NO_3_)]^5−^ clusters and their interactions with NH_4_^+^ under the applied potentials, three consecutive scans were performed. The nearly identical electrochemical responses and mass deposition/dissolution profiles throughout the cycling process (Figure S3a) corroborated the excellent reversibility of the electrolyte system. To quantitatively analyze the measured EQCM response, the obtained frequency shift was converted into Sauerbrey mass change (|Δm|) and correlated with the absolute charge passed (|Q|) during each cathodic and anodic sweep (Figure S4). To further correlate the mass evolution with the electrochemical charge transfer, the apparent molar mass per electron (M_app_) was calculated from the ratio of the areal mass change to the areal charge passed, as described in the Experimental Section. This parameter provides quantitative insight into the composition of the deposited phase and the role of NH_4_^+^ in charge compensation during deposition/dissolution process. For [V_18_O_46_(NO_3_)]^5−^ clusters in the presence of NH_4_^+^, the M_app_ values of 397.7, 449.2 and 437.8 g mol^−1^ e^−1^ were obtained for the three consecutive cathodic sweeps, while values of 454.9, 477.2 and 464.9 g mol^−1^ e^−1^ were obtained for the corresponding anodic sweeps. The average apparent molar masses are hence 428.2 g mol^−1^ e^−1^ for reduction/deposition process and 465.7 g mol^−1^ e^−1^ for the oxidation/dissolution process (Tables S1 and S2). These values are substantially larger than the molar mass of NH_4_^+^, suggesting that the measured mass change cannot be assigned solely to NH_4_^+^ transport. Instead, the dominant contribution originates from reversible reductive deposition and oxidative dissolution of a [V_18_O_46_(NO_3_)]^5−^‐derived phase accompanied by NH_4_^+^‐assisted charge compensation and possible solvent/electrolyte co‐transport. The Sauerbrey mass returns close to the initial baseline during anodic sweep, suggesting that the deposited POM‐containing film is largely removed together with the release of associated NH_4_^+^ species. Together, the EQCM analysis provides quantitative support for the proposed NH_4_^+^‐coupled [V_18_O_46_(NO_3_)]^−5^ deposition/dissolution mechanism.

Next, the electrodeposition behavior of the [PMo_12_O_40_]^3−^ cluster in the presence of NH_4_^+^ was investigated within a potential range from −800 to +500 mV (vs. Ag^+^/Ag), as shown in Figure 2b and Figure S3b. The first two reduction processes, observed at approximately 0 and −200 mV (vs. Ag^+^/Ag), exhibited insignificant mass variation, suggesting that these charge‐transfer reactions in this potential range mainly occurred in the solution phase. When the potential was lowered below −300 mV, a remarkable mass increase was observed, indicating that further interactions between the anionic POM species and NH_4_^+^ induced substantial mass deposition on the electrode surface. When the potential scan was reversed, the mass continued to increase in the first oxidation process, and subsequently, underwent a slow decrease in the second oxidation process and a rapid decrease in the third oxidation process. The final mass returned close to the initial value, indicating the reversible deposition/dissolution process of the [PMo_12_O_40_]^3−^ cluster in the presence of NH_4_^+^. The mass change during potential scan in the reduction direction showed that the [PMo_12_O_40_]^3−^ cluster tends to exhibit more complex electrochemical processes in the NH_4_PF_6_/MeCN solution involving both homogeneous solution‐phase redox reactions and heterogeneous deposition associated with a solution‐to‐solid phase transition over the entire potential range. Furthermore, the observed electrochemical response was quantified by correlating the Sauerbrey‐derived mass changes with the charge passed through cathodic and anodic sweeps. For the [PMo_12_O_40_]^3−^/NH_4_^+^ system, the analysis of the first three cathodic deposition cycles gave the M_app_ values of 591, 670, and 597 g mol^−1^ e^−1^, corresponding to the average M_app_ of 619 ± 44 g mol^−1^ e^−1^. This value is reasonably close to the theoretical value of 625.5 g mol^−1^ e^−1^, expected for an ammonium‐compensated PMo_12_‐derived deposit formed after uptake of three additional electrons by the initial [PMo_12_O_40_]^3−^ species, approximated as (NH_4_)_6_[PMo_12_O_40_]_red_. Therefore, the cathodic EQCM response supports the formation of an ammonium‐associated reduced PMo_12‐_derived film, where the mass increase is mainly attributed to PMo_12_‐based deposition accompanied by NH_4_^+^ charge compensation. However, the calculated anodic values were significantly higher and less reliable due to non‐rigid/viscoelastic contributions to the EQCM response (Figure S4). This anomalous anodic behavior, together with the pronounced changes in the full width at half maximum (FWHM), suggests that the deposited (NH_4_)_6_[PMo_12_O_40_]_red_ phase may be more porous, solvated or mechanically softer. During oxidation, electrolyte/solvent exchange, pore collapse, swelling/deswelling, or partial film restructuring may therefore contribute to the measured frequency response. Nevertheless, the electrochemical responses over the three consecutive scans show effective removal of the deposited film during anodic sweep within this potential window (Figure S3b), demonstrating the good reversibility of the system. When the cathodic potential was extended to value more negative than −800 mV (vs. Ag^+^/Ag), the deposited mass continued to increase; however, the process became irreversible, as evidenced by continuous mass accumulation on the electrode over multiple cycles (Figure S3c, d) [21].

Overall, the quantitative EQCM analyses established a direct correlation between electrochemical charge transfer and mass changes during the NH_4_^+^‐assisted POM deposition/dissolution. The calculated EQCM‐derived M_app_ shows that the [V_18_O_46_(NO_3_)]/NH_4_^+^ film exhibits highly consistent cathodic and anodic mass‐to‐charge correlation, indicative of a comparatively rigid and reversible deposit. In contrast, the [PMo_12_O_40_]/NH_4_^+^ process gives a cathodic M_app_ close to the expected ammonium‐compensated deposition process, but an anomalous anodic response that reflects increased viscoelastic contributions during oxidation and dissolution. Nevertheless, both POM systems undergo reversible NH_4_^+^‐coupled deposition/dissolution within appropriate potential windows, while the [PMo_12_O_40_]/NH_4_^+^‐derived deposits exhibit more complex dissolution dynamics than the corresponding [V_18_O_46_(NO_3_)]/NH_4_^+^‐derived deposits. In addition, complete mass dissolution could be achieved under sufficiently oxidative potentials, underscoring the critical importance of a suitable potential window for reversible solution‐to‐solid transitions.

### 2.3 Morphology and Compositional Analysis

**FIGURE 3:**
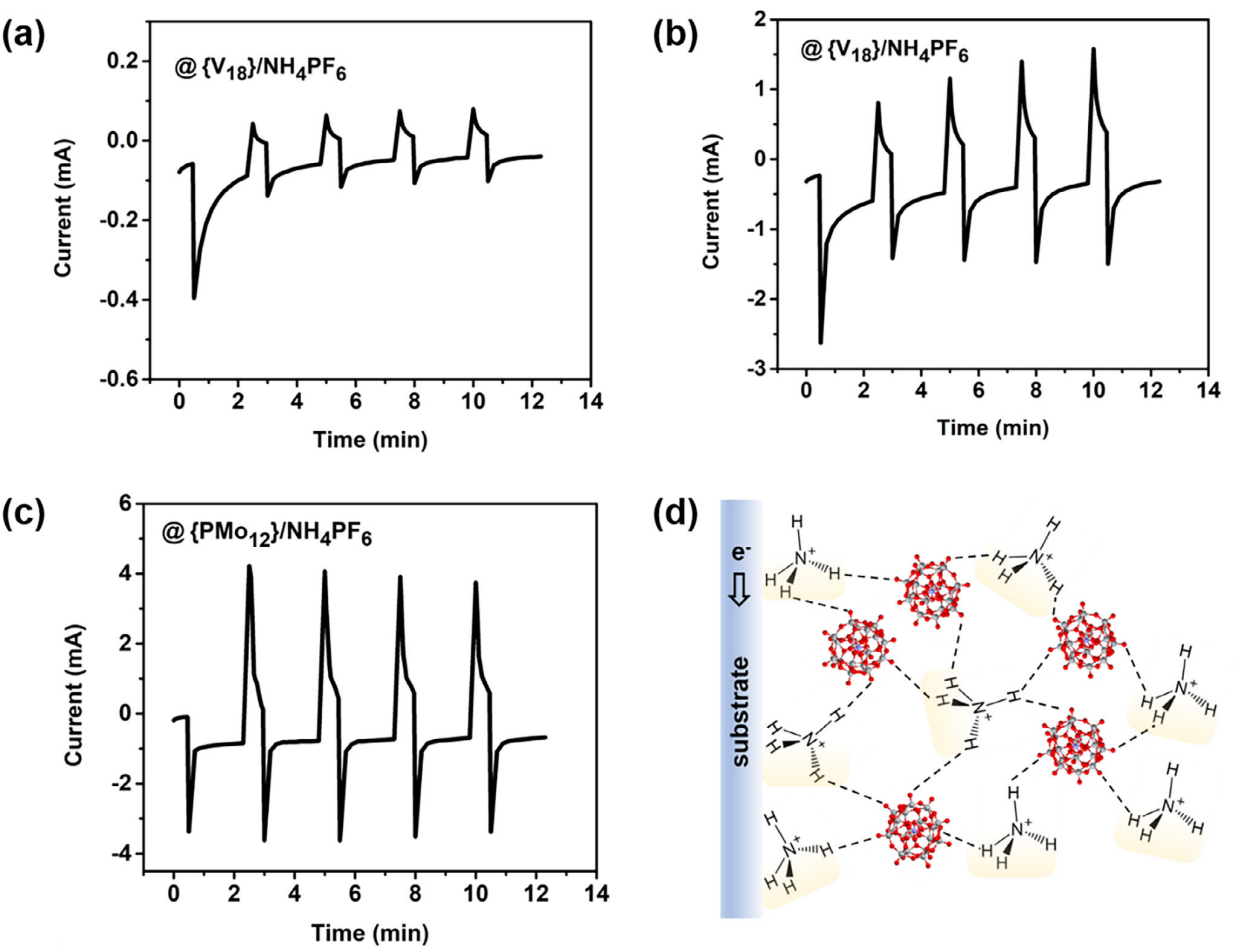
CSCA profiles of [V_18_O_46_(NO_3_)]^5−^ cluster in NH_4_PF_6_/MeCN reduced at (a) −300 mV and (b) −500 mV *vs*. Ag^+^/Ag; (c) CSCA profile of [PMo_12_O_40_]^3−^ cluster in NH_4_PF_6_/MeCN reduced at −800 mV *vs*. Ag^+^/Ag; (d) schematic illustration of proposed electron/NH_4_^+^ storage mechanism via POM‐derived electrodeposition on the substrate. Experimental conditions: anhydrous, de‐aerated acetonitrile containing concentrated NH_4_PF_6_ as supporting electrolyte, [V_18_O_46_(NO_3_)]^5−^ or [PMo_12_O_40_]^3−^ = 1 mm.

The electrodeposition from the NH_4_PF_6_/MeCN solution was also performed on the carbon‐coated aluminum foil (area 0.5 cm^2^) via the cyclic step chronoamperometry (CSCA) technique for further characterization. The deposition protocol consisted of alternating two periods for a total of five cycles, including a relaxation period at 0 mV (vs. Ag^+^/Ag) for 30 s, and a pulse period under a selected reduction potential for 120 s. The deposition from the [V_18_O_46_(NO_3_)]^5−^/NH_4_^+^ system was performed at −300 and −500 mV vs. Ag^+^/Ag, respectively (Figure 3a, b). The measured current during the pulse period at −500 mV was several times higher than that at −300 mV, indicating that electrodeposition process was highly potential‐dependent. The deposition from the [PMo_12_O_40_]^3−^/NH_4_^+^ system was performed at −800 and −1160 mV *vs*. Ag^+^/Ag (Figure 3c and Figure S5, respectively).

To elucidate the composition, electronic structure, and deposition mechanism of the NH_4_^+^‐coupled electrodeposits, the deposition products were investigated by scanning electron microscopy (SEM), energy‐dispersive x‐ray spectroscopy (EDX), x‐ray photoelectron spectroscopy (XPS), and attenuated total reflection Fourier‐transform infrared spectroscopy (ATR‐FTIR). The deposition from the [V_18_O_46_(NO_3_)]^5−^/NH_4_^+^ system at −300 mV (*vs*. Ag^+^/Ag) yielded a thin film‐like morphology with a thickness of approximately 1 µm, exhibiting noticeable surface cracking (Figure S6a,b). Elemental mapping via EDX revealed a homogeneous distribution of V, N, and O elements across the film, confirming that the deposited film was derived from the [V_18_O_46_(NO_3_)]^5−^ cluster (Figure S7a). By comparison, the deposition from the [PMo_12_O_40_]^3−^/NH_4_^+^ system at ‐1160 mV *vs*. Ag^+^/Ag resulted in a hierarchical nanoflake‐like structure (Figure S6 c, d). The corresponding EDX analysis showed a uniform distribution of Mo, P, and O elements, demonstrating that the film was derived from the pristine [PMo_12_O_40_]^3−^ cluster (Figure S7b) rather than from deposition of individual metal species. Despite originating from molecular POM precursors, the two deposited films exhibited markedly different morphologies. This suggests that the deposition pathway is strongly influenced by the intrinsic structure and reduction of the parent POM, consistent with EQCM results showing comparatively simple and highly reversible deposition/dissolution process for the [V_18_O_46_(NO_3_)]^5−^/NH_4_^+^ system, whereas the [PMo_12_O_40_]^3−^ cluster undergoes more complex electrochemical behavior involving both homogeneous solution‐phase redox reaction and heterogeneous deposition. Subsequently, the elemental composition and electronic states of the pristine POM clusters and the corresponding deposited products on the carbon‐coated Al foil were investigated by XPS spectroscopy (Figure S8). The obtained N 1s peak region was deconvoluted in detail since nitrogen‐containing species may originate from several possible sources, including [*n*Bu_4_N]^+^ counter cations, NH_4_^+^ from NH_4_PF_6_, nitrate, electrolyte residues or minor nitrogen‐containing surface species.

For the pristine [V_18_O_46_(NO_3_)]^5−^ cluster, the N 1s spectrum was deconvoluted into two components at approximately 402.0 and 406.3 eV, assigned to [*n*Bu_4_N]^+^ counter‐cation and nitrate template [25, 26], respectively (Figure S9). After electrodeposition from NH_4_PF_6_‐containing electrolyte, the deposited **{V_18_}**/NH_4_^+^ film exhibited an N 1s component in the 401–402 eV region, consistent with ammonium‐type nitrogen environment. However, this binding energy may overlap with [*n*Bu_4_N]^+^ and NH_4_^+^, the C 1s spectrum was also examined. The deposited film does not show a pronounced additional [*n*Bu_4_N]‐like carbon contribution beyond the carbon coated substrate/background, suggesting that residual [*n*Bu_4_N]^+^ is not the dominant nitrogen source. Moreover, the pristine [V_18_O_46_(NO_3_)]^5−^ cluster shows no F 1s signal, whereas the deposited film depicted only a weak F 1s signal, no detectable P 2p signal in the 125–150 eV region, indicating that significant intact NH_4_PF_6_/ PF_6_^−^ residue is unlikely (Figure S9). Next, in the pristine [V_18_O_46_(NO_3_)]^5−^ cluster (V^IV^_2_V^V^_16_), the V2p_1/2_ peak and V2p_3/2_ peak recorded at 523.5 and 516.3 eV, respectively, were attributed to V^IV^, and the V2p_1/2_ peak and V2p_3/2_ peak recorded at 524.8 and 517.5 eV, respectively, were attributed to V^V^ [19]. Fitting of the deconvoluted vanadium XPS spectrum indicates an atomic ratio of V^V^:V^IV^ of 15.9:2.1. In the deposited film, the V 2p_1/2_ peaks and V 2p_3/2_ peaks were retained, and the percentage contribution of V^IV^ increased to 13.7%, indicating a partial reduction of the vanadium centers. For the pristine [PMo_12_O_40_]^3−^ cluster, the characteristic Mo (3d_5/2_) and Mo (3d_3/2_) peaks at 233.0 and 236.1 eV, respectively, showed that all Mo elements were present in the +6 oxidation state (Figure S10) [16]. In the corresponding deposited product, in addition to the Mo^VI^ peaks, two new distinct Mo (3d_5/2_) and Mo (3d_3/2_) peaks at lower binding energies of 231.7 and 234.9 eV, respectively, were attributed to the presence of Mo^V^, suggesting the reduction of the Mo centers (Figure S10) [27]. The percentage of Mo^VI^ was approximately 34.2%, implying possible multi‐electron storage.

The ATR‐FTIR spectra of the pristine POM clusters and deposits were compared (Figure S11). In the IR spectra of the deposited products, the bending vibration at 1416 cm^−1^ was attributed to the N─H band [16, 27]. Since the N─H band was absent in the pristine POM clusters, this observation supported the uptake of NH_4_^+^ in the electrodeposition process. In addition, the downshifts of V = O_t_ and Mo = O_t_ stretching vibrations indicated the presence of hydrogen‐bonding interaction (N─H─O═M, where M is Mo or V) [16]. For the [PMo_12_O_40_]^3−^‐derived deposit, the two peaks at 3182 and 2995 cm^−1^ can be assigned to non‐hydrogen‐bonded and hydrogen‐bonded N─H stretching, respectively, which also supported the formation of hydrogen‐bonding interactions in the NH_4_^+^‐coupled deposit.

According to XPS and IR characterization results, during the electrodeposition process, the metal centers within the POM cluster frameworks were reduced, while charge‐compensating NH_4_^+^ migrated from the electrolyte solution into the deposited products. A hypothesized NH_4_^+^‐bridged vanadium oxide network based on [V_18_O_46_(NO_3_)]^5−^ in the initial electrodeposition process is illustrated in Figure 3d. Potential‐driven adsorption of NH_4_^+^ onto the substrate was followed by the interactions with the surrounding reduced POM clusters. In addition to electrostatic forces, NH_4_^+^ can engage in hydrogen‐bonding interactions with V‐O_x_ sites [9, 16]. As the nucleation and growth processes proceed, a deposited film is formed. The POM electrodeposition approach is facile, fast‐responsive and easily controllable. The complete dissolution of the deposited mass from the electrode surface shown in the EQCM characterization indicated the NH_4_^+^‐assisted POM electrodeposition/dissolution can be utilized in rechargeable energy‐storage devices for electron storage/release. The combined electrochemical, spectroscopic, and gravimetric data support a cation‐coupled electrodeposition mechanism fundamentally distinct from classical metal plating. Under reduction potentials, electron storage by the POM framework is accompanied by NH_4_^+^ uptake from the electrolyte to maintain electroneutrality. Unlike monoatomic cations, NH_4_^+^ additionally engages in directional hydrogen bonding with surface oxygen atoms of the POM framework, stabilizing the reduced molecular oxide network at the electrode surface. The combined electrostatic and hydrogen‐bonding interactions enable reversible formation of a solid phase that can be fully re‐oxidized and resolubilized, thereby realizing a molecular analogue of a deposition‐based charge‐storage system.

### 2.4 Deposition‐Based POM Hybrid Flow Battery

**FIGURE 4:**
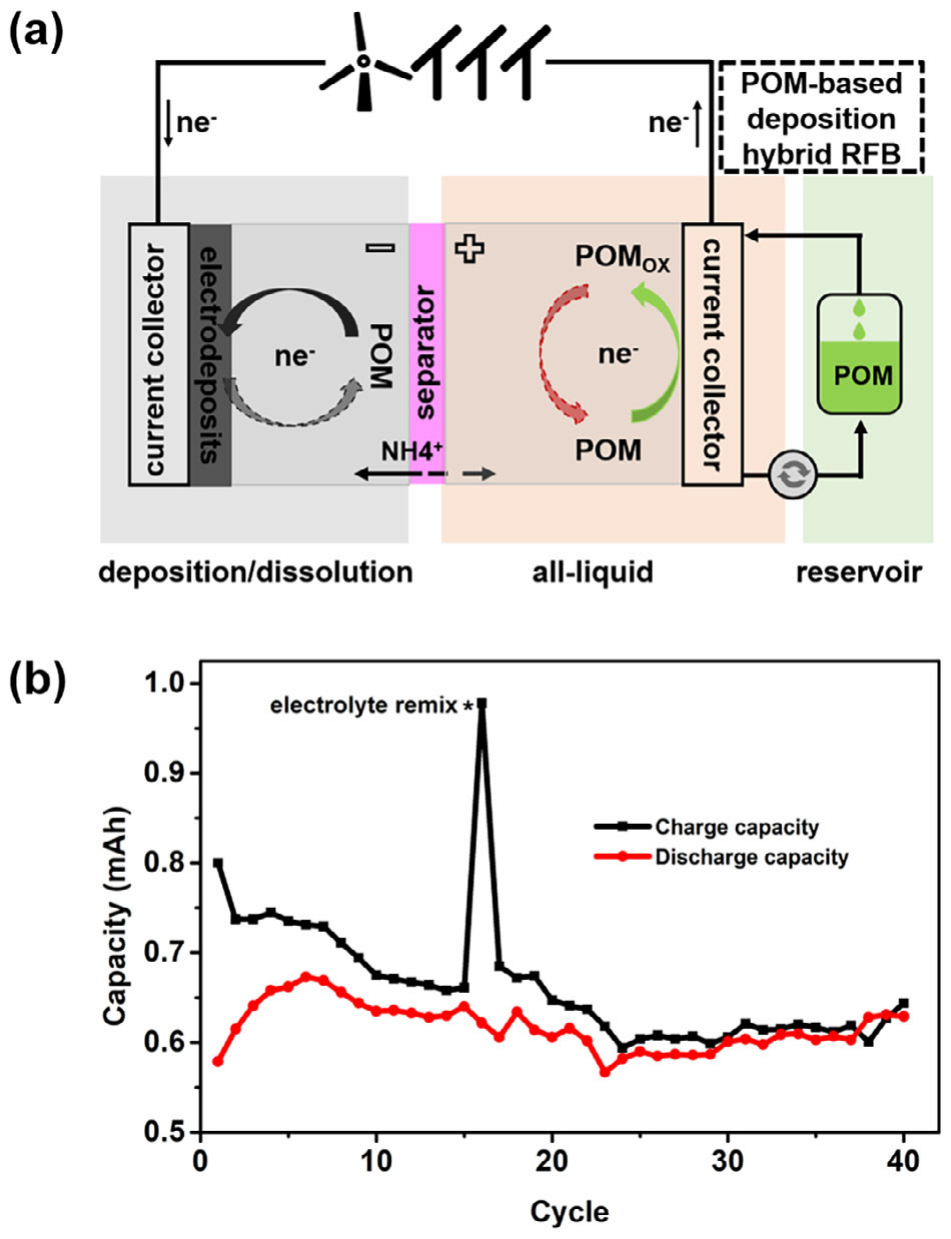
(a) Schematic illustration of deposition‐based POM hybrid RFBs; (b) cycling performance of the deposition‐based POM hybrid RFB using [V_18_O_46_(NO_3_)]^5−^ cluster as the redox material in the posolyte and negolyte.

To examine the feasibility of translating this reversible NH_4_^+^‐assisted deposition/dissolution behavior of the POM cluster, a proof‐of‐concept deposition‐based all‐POM hybrid RFB was investigated, as schematically illustrated in Figure 4a. During battery charging, the electrochemical reduction(s) in the negolyte lead to the NH_4_^+^‐coupled POM‐derived electrodeposit on the electrode in the negative half‐cell, while the POM clusters in the posolyte undergo oxidation reactions and remain dissolved. In the meantime, NH_4_^+^ cations transfer from the posolyte to the negative half‐cell through an ion‐exchange membrane to maintain charge balance. Upon discharging, the deposited film in the negative half‐cell is re‐oxidized and subsequently dissolved back into the electrolyte, while the oxidized POM clusters in the posolyte are reduced back to the pristine oxidation state. Accordingly, NH_4_^+^ is transferred from the negolyte to the positive half‐cell across the cell. A mixed‐valence POM cluster can be used as the same initial electrolyte for both positive and negative compartments for a deposition‐based hybrid RFB.

For the mixed‐valence [V_18_O_46_(NO_3_)]^5−^ cluster, the “open‐circuit potential” lies between the homogeneous solution redox reactions and heterogeneous solution‐to‐solid transition reactions, thus the cluster can be used as single redox material in the same initial electrolyte as both the posolyte and negolyte in a deposition‐based RFB. The cell was assembled using a piece of Nafion 117 membrane as an NH_4_^+^‐conducting separator for NH_4_^+^ transport [28, 29]. The pretreatment of the Nafion 117 membrane was performed based on a previously reported ammonium‐ion RFB [17], and the membrane was dried in vacuum at 80°C for 2d. Carbon felt was used as the electrode material in the positive half‐cell, while carbon paper were loaded between the membrane and the graphite current collector in the negative half‐cell.

The galvanostatic cycling performance of the [V_18_O_46_(NO_3_)]^5−^‐based hybrid RFB is shown in Figure 4b. The volume of the posolyte was twice that of the negolyte. The initial negolyte was charged until three electron storage and the cut‐off voltage was used in the following cycles. The cut‐off voltage was 0.02 V in the discharging process. During the initial cycles, the discharge capacity gradually increased, which is likely associated with progressive wetting and “activation” of the membrane and electrode surfaces in the ammonium‐containing nonaqueous electrolyte. Coulombic efficiency was above 90% indicating the Nafion 117 membrane as a suitable separator in the battery operation. The voltage efficiency was 68% and energy efficiency was around 61%. Upon continued cycling, a gradual capacity decay was observed, suggesting partial loss of electrochemically accessible active material in the negative half‐cell, likely caused by incomplete dissolution of the deposited POM phase or local accumulation of deposited species on the substrate surface. To evaluate the reversibility of the deposition‐based storage mechanism, a facile electrolyte remixing procedure was performed after fifteen cycles by transferring the posolyte into the negative half‐cell, followed by restoration of the initial electrolyte volume.

Following this treatment, the charging capacity recovered markedly and even exceeded the initial capacity. This pronounced recovery in charging capacity following electrolyte remixing suggests that capacity fading primarily originates from incomplete redissolution and local accumulation of electroactive POM deposits rather than irreversible molecular degradation. In the following cycles, the capacity gradually decreased again before reaching a relatively stable regime after approximately 25 cycles.

In addition to the V_18_‐based system, another deposition‐based POM hybrid RFB was also demonstrated using [V_18_O_46_(NO_3_)]^5−^ as redox species in the initial posolyte and [PMo_12_O_40_]^3−^ as redox species in the initial negolyte (Figure S12). The corresponding charge‐discharge profiles exhibited reproducible cycling behavior within a fixed operating potential window, confirming that distinct POM redox couples can be effectively integrated within the same NH_4_^+^‐mediated deposition‐based flow battery architecture. In particular, the use of the fully oxidized [PMo_12_O_40_]^3−^ cluster as the negolyte extends the accessible cathodic redox range, consistent with its deeper multi‐electron reduction behavior observed in the CV measurements, thereby broadening the electrochemical operating window of the asymmetric cell.

These results demonstrate that the NH_4_^+^‐assisted deposition‐dissolution mechanism is not restricted to single POM chemistry but can be extended to structurally distinct molecular metal oxide clusters with different redox properties. This highlights the versatility of the POM deposition‐based storage concept and provides a modular platform for tuning the voltage window, redox activity, and energy storage behavior through rational selection of posolyte and negolyte couples. Unlike metal deposition‐based batteries that rely on Mn^+^ ↔ M^0^ reactions, which frequently suffer from irreversible dendrite growth [30], the NH_4_^+^‐assisted POM electrodeposition occurs through reversible deposition and dissolution of molecular metal oxide species. Thus, NH_4_^+^‐assisted POM electrodeposition avoids metallic dendrite formation and enables a fundamentally different solution‐to‐solid charge storage pathway.

Overall, these proof‐of‐concept deposition‐based POM hybrid RFBs demonstrate that reversible NH_4_^+^‐assisted POM electrodeposition can be successfully integrated into a hybrid flow battery architecture, thereby broadening the design of hybrid redox flow batteries beyond conventional dissolved‐only or metal plating systems for electrochemical energy storage.

## 3 Conclusion

In this work, we demonstrate a facile NH_4_^+^‐assisted electrodeposition strategy for molecular metal oxides in a nonaqueous ammonium electrolyte solution. Using electrochemical, gravimetric, microscopic, and spectroscopic analyses, we reveal that polyoxometalate clusters undergo reversible reduction‐induced deposition and oxidation‐driven dissolution within a selected potential window, establishing a dynamic solution‐to‐solid charge‐storage mechanism. The electrodeposited phases exhibit coupled NH_4_^+^‐assisted electron storage, highlighting a fundamentally different behavior from conventional metal plating systems.

Building on this reversible deposition/dissolution chemistry, proof‐of‐concept POM‐based hybrid redox flow batteries were demonstrated, confirming the feasibility of integrating reversible POM electrodeposition into hybrid RFBs. This work establishes NH_4_^+^‐assisted reversible POM electrodeposition as a new deposition/dissolution mechanism for electrochemical energy storage and broadens the design of deposition‐based hybrid redox flow batteries beyond conventional dissolved‐only and metal‐deposition systems.

## 4 Experimental Methods

Detailed description of the methods and instruments used in this work is provided in the Supporting Information

## Author Contributions

**Ke Wang**: methodology, validation, writing – original draft, Writing – review and editing, formal analysis, data curation, resources, investigation. **Nikhil Arya**: investigation, methodology, writing – review and editing, formal analysis, resources. **Sadia Shahid**: investigation, writing – review and editing, formal analysis, methodology. **Montaha Anjass**: conceptualization, methodology, validation, writing – review and editing, funding acquisition, investigation, project administration, resources, supervision, data curation.

## Conflicts of Interest

The authors declare no conflicts of interest.

## Supporting information




**Supporting File**: advs77673‐sup‐0001‐SuppMat.docx.

## Data Availability

The data that support the findings of this study are available in the supplementary material of this article.
