# Supplementary material for: Potential‐Controlled Ammonium‐Assisted Reversible Electrodeposition of Polyoxometalates for Hybrid Redox Flow Batteries
Source: Adv Sci (Weinh). 2026 Sep 16:e77673. Online ahead of print. doi: 10.1002/advs.77673 (PMC13583220; doi:10.1002/advs.77673)
Supplement: Supplementary file 1 [file ADVS-9999-e77673-s001.docx]

Electronic Supporting Information

**Potential-Controlled Ammonium-Assisted Reversible Electrodeposition of Polyoxometalates for Hybrid Redox Flow Batteries**

*Ke Wang, Nikhil Arya, Sadia Shahid, and Montaha Anjass*

1. **Materials**

Acetonitrile (Acroseal®, 99.9 %, Extra Dry), ammonium hexafluorophosphate (Sigma, 99.98%), sodium phosphomolybdate (Sigma, technical grade), carbon felt (SGL), carbon paper (Toray), and Nafion 117 (Dupont) were used. All other chemicals were of reagent grade and used without further purification unless stated otherwise.

**Synthesis of (*n*Bu_4_N)_5_[V_18_O_46_(NO_3_)]{V_18_}:** **{V_18_}** was synthesized according to previously reported procedures ^[13]^. Briefly, (*n*Bu_4_N)_3_[H_3_V_10_O_28_] was heated at 195^o^C for two hours under air conditions at a heating rate of 200 K h^‑1^. until the color changed from orange to dark brown. After cooling, the powder (0.4 g, 0.29 mmol, 1 eq.) was dissolved in 40 mL acetonitrile, then (*n*Bu_4_N)NO_3_ (1.6 g, 5.26 mmol, 18 eq.) was added. The mixture was stirred overnight at room temperature and then filtered. The dark brown crystals were obtained after a couple of days of diffusion of ethyl acetate into the filtered solution.

**Synthesis of (*n*Bu_4_N)_3_[PMo_12_O_40_]:** Na_3_[PMo_12_O_40_] xH_2_O (1.87 g, 1 mmol, 1 eq.) was dissolved in deionized water, and then (*n*Bu_4_N)Cl (1.39 g, 5 mmol, 5 eq.) was added. After stirring overnight and filtration, the solids were washed several times with water, dried overnight in the fume hood, and stored under vacuum in a desiccator for two days.

2. **Methods and Instrumentation**

**Attenuated total reflection-Fourier transform infrared spectroscopy (ATR-FTIR)** was performed using a Bruker Alpha II equipped with an ATR Platinum Diamond unit. All spectra were recorded at room temperature in a spectral range from 4000 cm^-1^ to 400 cm^-1^.

**X-ray photoelectron spectroscopy (XPS)** was performed on a Physical Electronics PHI 5800 spectrometer with monochromatized Al_Kα_ (1486.6 eV) radiation. The measurements were performed with a detection angle of 45, using pass energies at the analyzer of 93.9 and 29.35 eV for survey and detailed spectra, respectively. The samples were neutralized with electrons from a flood gun (current: 3 µA) to compensate for surface charging effects.

**Scanning Electron Microscopy (SEM) and Energy-dispersive X-ray spectroscopy (EDX)** were performed on a Zeiss Gemini Leo 1550 VP equipped with a Silicon Drift Detector (OXFORD Instruments) using a 10 keV electron beam. The deposited material was rinsed with acetonitrile to remove residual species before further investigation.

**Electrochemical characterization:** Cyclic voltammetry was performed using a Pine WaveDriver 200 potentiostat/galvanostat with IR-compensation set to 95%. The uncompensated resistance was determined by impedance spectroscopy using the EIS-RU tool. The concentrated NH_4_PF_6_/MeCN solution was obtained by the filtration of oversaturated NH_4_PF_6_/MeCN solution through a PTFE syringe filter. The concentration of the saturated electrolyte was determined gravimetrically. A total of 1.8925 g of NH_4_PF_6_ was initially added to 20 ml of anhydrous MeCN, while 98.35 mg of undissolved NH_4_PF_6_ was recovered after filtration. Accordingly, 1.7942 g of NH_4_PF_6_ remained dissolved in 20.00 ml MeCN, corresponding to 0.55 M. The analytes were prepared by dissolving the POM cluster (1 mM) in the concentrated NH_4_PF_6_/MeCN solution. The solutions were purged with argon for at least 15 min to remove O_2_ prior to experiments, and kept under quasi-argon atmosphere during the whole measurements. The cyclic voltammetry was conducted in a typical three-electrode cell, with a glassy carbon (diameter 3 mm) as working electrode, a platinum wire as counter electrode. A silver wire immersed in the concentrated NH_4_PF_6_/MeCN solution in a glass frit was used as a quasi-reference electrode. Scan rate is 50 mV s^-1^.

**Cyclic Step Chronoamperometric method of [V_18_O_46_(NO_3_)]^5-^ and [PMo_12_O_40_]^3-^:** Pulsed electrodeposition (PED) was performed via a Pine WaveDriver 200 potentiostat/galvanostat with the cyclic step chronoamperometry (CSCA) program via a H-type glass cell. The analyte solution was loaded into the working half-cell, in which a carbon-coated aluminum foil was used as working electrode with the immersed area of 0.5 cm^2^. A silver wire immersed in the concentrated NH_4_PF_6_/MeCN solution in a glass frit was used as a quasi-reference electrode. Blank NH_4_PF_6_/MeCN solution was loaded into the other half-cell, in which carbon felt was used as counter electrode. The CSCA experiment was performed by two distinct steps: 0 mV (*vs.* Ag/Ag^+^) was initially applied to the working electrode for 30 seconds, then a selected reduction potential was applied for a period of 120 seconds. These two steps were repeated alternately for five successive iterations.

**Electrochemical quartz crystal microbalance:** EQCM experiments were performed with a CHI440 electrochemical quartz crystal microbalance (CH Instruments, TX, USA) using 5 MHz quartz crystal sensors with Au-coating (CH Instruments, TX, USA) in a three-electrode setup: Au coated quartz crystal as working electrode, a silver wire as reference electrode and a platinum wire as a counter electrode.

**Quantitative EQCM analysis (apparent mass per-electron value M_app_):** The EQCM response was quantitatively analysed by plotting the Sauerbrey mass change against	the charge passed during POMs deposition and dissolution. The resulting slope was used to calculate the apparent mass per-electron value (M_app_). The EQCM frequency response also affected by the physical properties of the deposited film like film’s rigidity, viscosity, roughness and solvent coupling, the calculated values are discussed as M_app_ values.^[1]^

The given Sauerbrey equation related to the frequency shift of the quartz crystal used to the areal mass change:

Δm_A_ = -C_f_ $\frac{\Delta f}{n}$

Where Δm_A_ is Sauerbrey areal mass changes in ng cm^-2^_,_ C_f_ is the sensitive constant of the quartz crystal Δf is the frequency shift in Hz, and n is the overtone number. In this work the n=1, the Sauerbrey mass directly given by the EQCM instrument in ng cm^-2^.

For a 5 MHz crystal, the approximate Sauerbrey constant is calculated as:

C_f_ = ~ 17.7 ng cm^-2^ Hz^-1^

A negative frequency shift corresponds to a positive mass increase at the electrode surface.

The electrochemical charge passed during each cyclic voltammetry sweep was obtained by integrating the current over time:

Q =$\int I\left( t \right)dt$

Where Q = charge is the charge (C), I(t) is the current in (A) and t is the time, the charge was normalized by the active electrode area A=πr^2^ = 1.13 cm^2^.

Finally, the M_app_ determined by the form of the slope of the Sauerbrey mass change versus charge plot:

Δm_A_ = SQ + b

Where S=( Δm_A_/ ΔQ_A_) is the slope of the mass-charge plot. If Δm_A_ is expressed in g cm^-2^ and ΔQ_A_ in C cm^-2^

The M_app_ was then calculated using the Faraday’s constant:

M_app_ = F × $\left| \Delta mA/ \Delta QA \right|$

Where F is 96485 C mol^-1^ and S=( Δm_A_/ ΔQ_A_) is the slop of the linear fit of Δm_A_ vs. Q_A._

**Redox flow battery:** RFB tests were performed with a LAND battery testing system. Graphite plate was used as a current collector, and the electrode area was 7.29 cm^2^. Nafion 117 membrane was used as a separator with a pretreatment process: the membrane was immersed in 3% H_2_O_2_ solution at 80 °C for 1 h, and then rinsed with deionized water; subsequently, it was immersed in 0.2 M NH_4_PF_6_/H_2_O for 1 d and dried at 80 °C in vacuum for 2 d. Prior to battery test, the pretreated Nafion 117 was immersed in concentrated NH_4_PF_6_/anhydrous MeCN solution for 1 d. A piece of carbon felt was used as the positive electrode. In the negative half-cell, five pieces of carbon paper were placed between the Nafion 117 membrane and a graphite plate.

In the [V_18_O_46_(NO_3_)]^5-^-based symmetric hybrid flow battery, the posolyte was 20 mL of 1 mM [V_18_O_46_(NO_3_)]^5-^ in concentrated NH_4_PF_6_/MeCN solution, and the negolyte was 10 mL of 1 mM [V_18_O_46_(NO_3_)]^5-^ in concentrated NH_4_PF_6_/MeCN solution. The posolyte and negolyte were circulated with flow rate of around 20 ml/min by a peristaltic pump. Charging and discharging current was 0.146 mA. The first charging process was operated until the capacity reached 0.8 mAh (corresponding to three electrons transfer in the negolyte), and the voltage was used as cut-off voltage for the following cycles. The cut-off voltage of the discharging process was set as 0.02 V.

In the [V_18_O_46_(NO_3_)]^5-^//[PMo_12_O_40_]^3-^ hybrid flow battery, the posolyte and negolyte were 1 mM [V_18_O_46_(NO_3_)]^5-^ and [PMo_12_O_40_]^3-^ in concentrated NH_4_PF_6_/MeCN solution, respectively. The posolyte was 20 mL of 1.5 mM [V_18_O_46_(NO_3_)]^5-^ in concentrated NH_4_PF_6_/MeCN solution, and the negolyte was 10 mL of 0.5 mM [PMo_12_O_40_]^3-^ in concentrated NH_4_PF_6_/MeCN solution. The charging and discharging current was 0.146 mA. The initial charging process was operated until the capacity reached to 0.804 mAh (six electrons transfer in the negolyte), and the resulting voltage was used as the cut-off voltage for subsequent cycles. The cut-off voltage of the discharging process was set to 0.01 V.

3. **Cyclic voltammetry of {V_18_} and {PMo_12_} clusters**

**Figure S1.** Cyclic voltammograms of **a)** [V_18_O_46_(NO_3_)]^5-^ and **b)** [PMo_12_O_40_]^3-^ in (*n*Bu_4_N)PF_6_/MeCN solution (experimental conditions: anhydrous, de-aerated acetonitrile containing 0.1 M (*n*Bu_4_N)PF_6_ as supporting salt, [V_18_O_46_(NO_3_)]^5-^ or [PMo_12_O_40_]^3-^ = 1 mM; CV scan rate: 100 mV s^‑1^).

**Figure S2.** Cyclic voltammograms of [PMo_12_O_40_]^3-^ in NH_4_PF_6_/MeCN solution (experimental conditions: anhydrous, de-aerated acetonitrile containing concentrated NH_4_PF_6_ as supporting salt, [PMo_12_O_40_]^3-^ = 1 mM; CV scan rate: 50 mV s^‑1^).

4. **a) EQCM measurement**

**Figure S3**: EQCM measurement of **a)** [V_18_O_46_(NO_3_)]^5-^ in NH_4_PF_6_/MeCN between -500 and 1300 mV (vs. Ag^+^/Ag), and [PMo_12_O_40_]^3-^ in NH_4_PF_6_/MeCN **b)** between -800 and 500 mV (vs. Ag^+^/Ag) and **c)** between -1500 and 300 mV (vs. Ag^+^/Ag) (experimental condition: anhydrous, de-aerated acetonitrile containing concentrated NH_4_PF_6_ as supporting salt, [V_18_O_46_(NO_3_)]^5-^ or [PMo_12_O_40_]^3-^ = 0.5 mM while using cyclic voltammetry at a scan rate of 30 mV s^-1^); **d)** digital photos of [PMo_12_O_40_]^3-^ electrolyte after the EQCM measurement in the wide potential window, as well as the deposits on the working electrode surface.

4. **b) Quantitative EQCM analysis and apparent mass-per-electron (M_app_) calculation**

**Figure S4**: Normalized correlation between the absolute Sauerbrey mass change, |Δm| and the absolute charge passed,(|Q|), for the NH_4_^+^-assisted deposition and dissolution of the derived POM films. **a)** [V_18_O_46_(NO_3_)]^5-^ /NH_4_^+^ and **b)** [PMo_12_O_40_]^3-^ /NH_4_^+^. Cathodic sweeps represent the deposition of the POM-containing film, while the anodic sweeps correspond to oxidative dissolution/extraction of the deposited film. The endpoint lines were normalized to |Q|= 1 mC cm^-1^. The resulting slopes were used to calculate the M_app_, confirming that the mass response arises from POM-film deposition/dissolution coupled with NH_4_^+^ assisted charge compensation rather than NH_4_^+^ transport alone.

**Table S1**. EQCM -derived M_app_ and apparent electron number calculated from |Δm| vs. |ΔQ| plot for the cathodic deposition and anodic dissolution of [V_18_O_46_(NO_3_)]^5-^/NH_4_^+^ and [PMo_12_O_40_]^3-^/NH_4_^+^ films during the first cycle.

| **System** | **Segment** | **Slope (ng mC⁻¹)** | **M_app_**  **(g mol^-1^ e^-1^)** | **M_film_**  **(g mol⁻¹)** | **Apparent (n)** |
| --- | --- | --- | --- | --- | --- |
| V₁₈/ NH₄⁺ | Cathodic deposition | 4121.5326 | 397.7 | 1805.1 | 4.54 |
| V₁₈/ NH₄⁺ | Anodic dissolution | 4714.7835 | 454.9 | 1805.1 | 3.96 |
| PMo₁₂/ NH₄⁺ | Cathodic deposition | 6127.4 | 591.2 | 1930.56 | 3.26 |
| PMo₁₂/ NH₄⁺ | Anodic dissolution | 22439 | 2165.1 | 1930.56 | 0.89 |

Here the anomalously high anodic M_app_ value for the PMo_12_/NH_4_^+^ system is not used for stoichiometric assignment because the anodic EQCM response is strongly affected by non-rigid/viscoelastic contributions, likely arising from film porosity, solvent/electrolyte exchange, swelling/deswelling, and partial film restructuring during oxidative dissolution. Therefore, the apparent electron number for the anodic PMo_12_ process is shown only as a mathematical value and should not be interpreted as a physically meaningful electron-transfer number.

**Table S2**. Theoretical M_app_ values estimated from the assumed ammonium-containing POM film components and expected electron numbers for [V_18_O_46_(NO_3_)]^5-^/NH_4_^+^ and [PMo_12_O_40_]^3-^/NH_4_^+^ system.

| **System** | **Assumed film composition** | **Molecular mass ( g mol⁻¹)** | **Expected e⁻** | **Theoretical M_app_ ( g mol⁻¹ e⁻¹)** |
| --- | --- | --- | --- | --- |
| {V₁₈}/ NH₄⁺ | (NH_4_)_5_ [V_18_O_46_(NO_3_)] | 1805.1 | 5 | 361.0 |
| {PMo₁₂}/ NH₄⁺ | (NH_4_)_6_ [PMo_12_O_40_]_red_ | 1930.6 | 3 | 643.5 |

5. **CSCA measurement**

**Figure S5**: CSCA profile of [PMo_12_O_40_]^3-^ cluster at reduction potential of -1160 mV (vs. Ag^+^/Ag) in a single cell (experimental condition: anhydrous, de-aerated acetonitrile containing concentrated NH_4_PF_6_ as supporting salt, [PMo_12_O_40_]^3-^ = 1 mM).

6. **Scanning Electron Microscopy and Energy-dispersive X-ray spectroscopy**

**Figure S6**. **a)** and **b)** top view of [V_18_O_46_(NO_3_)]^5-^/NH_4_^+^-derived film deposited at -300 mV (vs. Ag^+^/Ag); **c)** and **d)** top view of [PMo_12_O_40_]^3-^/NH_4_^+^-derived film deposited at -1160 mV (vs. Ag^+^/Ag).

**Figure S7.** SEM image (left), EDX elemental mapping (middle) and EDX spectra (lright) of **a)** [V_18_O_46_(NO_3_)]^5-^/NH_4_^+^-derived film deposited at -300 mV (vs. Ag^+^/Ag); **b)** [PMo_12_O_40_]^3--^/NH_4_^+^-derived film deposited at -1160 mV (vs. Ag^+^/Ag).

7. **X-ray photoelectron spectroscopy**

**Figure S8.** Full XPS spectra of **a)** [V_18_O_46_(NO_3_)]^5-^ and [PMo_12_O_40_]^3-^ clusters, and **b)** [V_18_O_46_(NO_3_)]^5-^/NH_4_^+^-derived film deposited at -300 mV (vs. Ag^+^/Ag), and [PMo_12_O_40_]^3-^/NH_4_^+^-derived film deposited at -1160 mV (vs. Ag^+^/Ag), respectively.

**Figure S9.** High-resolution XPS spectra of pristine [V_18_O_46_(NO_3_)]^5-^ cluster in **a)** O 1s/V 2p region and **b)** N 1s region, and [V_18_O_46_(NO_3_)]^5-^/NH_4_^+^-derived film deposited at -300 mV (vs. Ag/Ag^+^) in **c)** O 1s/V 2p region and **d)** N 1s region, respectively. Carbon 1s spectra of **e)** [V_18_O_46_(NO_3_)]^5-^/NH_4_^+^-derived film and **f)** pristine [V_18_O_46_(NO_3_)]^5-^ cluster. g) F 1s spectrum of [V_18_O_46_(NO_3_)]^5-^/NH_4_^+^ derived film.

**Figure S10.** High-resolution XPS spectrum of the pristine [PMo_12_O_40_]^3-^cluster in **a)** Mo 3d region **b)** N 1s region, and high-resolution XPS spectrum of [PMo_12_O_40_]^3-^/NH_4_^+^-derived film deposited at -1160 mV (vs. Ag/Ag^+^) in **c)** Mo 3d region, **d)** N 1s region,

8. **Infrared spectroscopy (ATR-FTIR)**

**Figure S11.** ATR-FTIR spectra in the spectral range from 3500 cm^-1^ to 400 cm^-1^ of (a) aluminum foil, (b) carbon-coated aluminum foil, (c) [V_18_O_46_(NO_3_)]^5-^ cluster, (d) [PMo_12_O_40_]^3-^ cluster, (e) [V_18_O_46_(NO_3_)]^5-^/NH_4_^+^ deposited film at -300 mV (*vs.* Ag/Ag^+^) onto carbon-coated aluminum foil, (f) [PMo_12_O_40_]^3-^/NH_4_^+^ deposited film at -800 mV (*vs.* Ag/Ag^+^) onto carbon-coated aluminum foil.

9. **Deposition-based [V_18_O_46_(NO_3_)]^5-^//[PMo_12_O_40_]^3-^ flow battery**

**Figure S12.** Cycling performance of [V_18_O_46_(NO_3_)]^5-^//[PMo_12_O_40_]^3-^ RFB.

10. **Author Contributions**

Authors’ Contribution statement using CRediT with the degree of contribution (<https://authorservices.wiley.com/author-resources/Journal-Authors/open-access/credit.html>)

K. Wang: Development of the methodology, validation, formal analysis, writing – the original draft (lead), data curation, writing – review & editing, investigation, and visualization; N. Arya: Resources, formal analysis (performed EQCM, SEM/EDX), and writing – review & editing; S. Shahid: Resources, formal analysis (performed electrochemistry), and writing – review & editing; Montaha Anjass: Conceptualization, development of the methodology, resources, writing - review & editing, supervision, project administration, funding acquisition. All authors analyzed data, revised the manuscript and have approved the final version of the article.

**References**

[1] M. D. Levi, N. Levy, S. Sigalov, G. Salitra, D. Aurbach, and J. Maier, Electrochemical quartz crystal microbalance (EQCM) studies of ions and solvents insertion into highly porous activated carbons. *J. Am. Chem. Soc.* **2010***,* *132*, 13220-13222.
